# Evaluating the efficacy of large language models in cardio-oncology patient education: a comparative analysis of accuracy, readability, and prompt engineering strategies

**DOI:** 10.3389/frai.2025.1693446

**Published:** 2026-01-13

**Authors:** Zhao Wang, Lin Liang, Hao Xu, Yuhui Huang, Chen He, Weiran Xu, Haojie Zhu

**Affiliations:** 1 Department of Cardiology and Institute of Vascular Medicine, Peking University Third Hospital, Beijing, China; 2 Department of Cardiology, Shanghai General Hospital, Shanghai Jiao Tong University School of Medicine, Shanghai, China; 3 Cardiac Arrhythmia Center, Fuwai Hospital, National Center for Cardiovascular Diseases, Peking Union Medical College and Chinese Academy of Medical Sciences, Beijing, China; 4 Department of Medical Oncology, Beijing Tiantan Hospital, Capital Medical University, Beijing, China

**Keywords:** artificial intelligence, cardio-oncology, large language models, patient education, prompt engineering

## Abstract

**Background:**

The integration of large language models (LLMs) into cardio-oncology patient education holds promise for addressing the critical gap in accessible, accurate, and patient-friendly information. However, the performance of publicly available LLMs in this specialized domain remains underexplored.

**Objectives:**

This study evaluates the performance of three LLMs (ChatGPT-4, Kimi, DouBao) act as assistants for physicians in cardio-oncology patient education and examines the impact of prompt engineering on response quality.

**Methods:**

Twenty standardized questions spanning cardio-oncology topics were posed twice to three LLMs (ChatGPT-4, Kimi, DouBao): once without prompts and once with a directive to simplify language, generating 240 responses. These responses were evaluated by four cardio-oncology specialists for accuracy, comprehensiveness, helpfulness, and practicality. Readability and complexity were assessed using a Chinese text analysis framework.

**Results:**

Among 240 responses, 63.3% were rated “correct,” 35.0% “partially correct,” and 1.7% “incorrect.” No significant differences in accuracy were observed between models (*p* = 0.26). Kimi demonstrated no incorrect responses. Significant declines in comprehensiveness (*p* = 0.03) and helpfulness (*p* < 0.01) occurred post-prompt, particularly for DouBao (accuracy: 57.5% vs. 7.5%, *p* < 0.01). Readability metrics (readability age, difficulty score, total word count, sentence length) showed no inter-model differences, but prompts reduced complexity (e.g., DouBao’s readability age decreased from 12.9 ± 0.8 to 10.1 ± 1.2 years, *p* < 0.01).

**Conclusion:**

Publicly available LLMs provide largely accurate responses to cardio-oncology questions, yet their utility is constrained by inconsistent comprehensiveness and sensitivity to prompt design. While simplifying language improves readability, it risks compromising clinical relevance. Tailored fine-tuning and specialized evaluation frameworks are essential to optimize LLMs for patient education in cardio-oncology.

## 1 Introduction

According to the latest GLOBOCAN 2022 data released by the International Agency for Research on Cancer (IARC), approximately 19.96 million new cancer cases and 9.73 million cancer-related deaths were reported globally in 2022 (Bray et al., 2024). Meanwhile, significant advancements in the early detection and treatment of cancer have led to a growing population of cancer survivors worldwide (Verdecchia et al., 2007; de Moor et al., 2013).

Cancer and cardiovascular diseases remain among the leading causes of morbidity and mortality globally. In recent years, the interdisciplinary field of cardio-oncology has emerged, addressing the intersection of these two major health challenges. Population aging, coupled with prolonged survival resulting from improved anti-tumor therapies, has contributed to a substantial increase in the number of patients with coexisting cancer and cardiovascular diseases. These conditions often share common high-risk factors, including unhealthy smoking habits, poor diet, and physical inactivity (Zheng et al., 2023). Furthermore, malignant tumors and their treatments—such as chemotherapy and radiotherapy—can trigger or worsen cardiac damage. Broadly, primary cardiac tumors are also encompassed within the scope of cardio-oncology.

The comprehensive management of diseases in the field of cardio-oncology is closely linked to patients’ understanding of their condition and their lifestyle habits. However, due to the interdisciplinary and complex nature of this field, there is a notable lack of patient education resources. This gap between patients’ informational needs and the content currently available significantly impacts adherence and treatment outcomes. Providing patients with professional and accurate information on cardio-oncology is therefore crucial.

In recent years, artificial intelligence (AI) has made significant advancements. Notably, the use of natural language processing (NLP) technology has made it possible to digitally represent text through word embeddings. This enables large-scale medical text data to be utilized by neural networks for end-to-end medical education, healthcare services, and other applications, such as medical consultation chatbots (An et al., 2024). AI-powered chatbots, such as ChatGPT, have demonstrated the potential to provide reliable, accessible, and personalized information, significantly improving patient education and the overall disease experience (Cascella et al., 2023). With its latest iteration, ChatGPT-4, AI language models are now capable of responding to a wide range of health-related questions and topics (Xie et al., 2023; Seth et al., 2023). In China, large language models trained on Chinese corpora, such as Kimi and DOUBAO, are also being widely applied in the field of health education (Li, 2024).

Since these large language models (LLMs) are accessible to the general public, including patients, it is essential to explore their performance in specialized fields such as cardio-oncology. However, due to the high demand for accuracy and low tolerance for error in this domain, current LLMs are not yet suitable for independently addressing medical professional questions. In this study, we designed a scenario where LLMs act as assistants for physicians in patient education, specifically aiding in the creation of educational materials related to cardio-oncology. Physicians then evaluate the outputs of the models for accuracy, safety, and other critical aspects to validate their performance in professional applications. Additionally, we utilized Chinese text analysis tools to comprehensively evaluate the readability and complexity of response. This study also compared the performance of different LLMs in addressing questions across various subfields within cardio-oncology and explored strategies for prompt design. The findings provide valuable insights into the application of LLMs in cardio-oncology and lay a foundation for the development of specialized patient education tools in this field. Therefore, this study aims to answer the research question: How do three publicly available LLM-based chatbots perform in responding to common questions on cardio-oncology and how do prompt influence LLM-based chatbots performance.

## 2 Methods

We investigate the utility of three publicly available and popular chatbot, ChatGPT-4, Kimi and DouBao, as an educational resource for patients on cardio-oncology. The three chatbots evaluated in this study were the publicly available versions from their respective developers, accessed via their web interfaces on October 21, 2024. Specific details are as follows: ChatGPT-4 (OpenAI, version via https://chat.openai.com/), Kimi (Moonshot AI, version via https://www.kimi.com/) and DouBao (ByteDance, version via https://www.doubao.com/chat/). As public chat interfaces were used, the models’ hyperparameters were set to their default, non-adjustable configurations, reflecting a typical “out-of-the-box” user experience. Each chatbot was queried two times with an identical set of 20 sequential questions pertaining to patient education on cardio-oncology. The 20 questions were developed based on 2022 ESC Guidelines on cardio-oncology and frequently asked questions (FAQs) from clinical practice. To ensure these questions covered various aspects of cardio-oncology, we invited two attending physicians specializing in this field to discuss and review, and finally determined these 20 most common questions in patient education for cardio-oncology (Table 1). And first, the questions were submitted to each publicly accessible AI chatbot through its online portal. Second, the questions were relayed to each chatbot, with the subsequent prompt used before each question: “*Please answer the following question in the most straightforward and easy-to-understand language: (…)*.” For each question, a new window of the respective chatbot was created to avoid any biases from the prior questions. After the answers were generated, they were recorded verbatim in our database. All questions were relayed to each chatbot in Chinese.

**Table 1:** Question list.

Number	Question
1	What is the definition of cancer therapy-related cardiovascular toxicity?
2	What are the risk factors of cancer therapy-related cardiovascular toxicity?
3	What are the types of cancer therapy-related cardiovascular toxicity?
4	Which cancer therapy can lead to cardiovascular toxicity?
5	Can cancer patients with underlying heart disease receive chemotherapy, radiation therapy, or targeted therapy?
6	Can patients with heart failure receive bone marrow transplantation?
7	How to prevent cancer therapy-related cardiovascular toxicity?
8	What tests are needed to diagnose cancer therapy-related cardiovascular toxicity?
9	When do cancer patients with cardiovascular toxicity need myocardial biopsy?
10	When do cancer patients with cardiovascular toxicity need coronary angiography or coronary CTA?
11	How to conduct cardiac monitoring (frequency and items) for cancer patients with cardiovascular toxicity?
12	Which should be treated first, cancer or cardiovascular toxicity?
13	Do cancer patients with cardiovascular toxicity have to stop cancer treatment?
14	When should cancer patients with cardiovascular toxicity stop tumor treatment/switch tumor treatment plans?
15	Can cancer patients with cardiovascular toxicity be cured?
16	Which doctors should cancer patients with cardiovascular toxicity seek treatment from?
17	After completing cancer treatment, is it still necessary to have regular heart checkups?
18	What is the incidence rate of cardiac tumors?
19	What is the survival time and prognosis of cardiac amyloidosis?
20	Can cardiac amyloidosis be inherited?

Answers were reviewed and graded based on accuracy, comprehensiveness, helpfulness, and practicality. Accuracy, comprehensiveness, and helpfulness were each graded into three levels, while practicality was measured by the question: “*Would you use this answer for patient education?*” All responses were independently evaluated by four specialists: two attending oncologists and two cardio-oncology subspecialists. All evaluators had over 3 years of post-fellowship experience in their respective fields. Response accuracy was guided by preexisting published literature or guidelines.

For each answer, we also investigate its readability and complexity using a previously published Chinese text analysis framework (Cheng et al., 2020), including four dimensions: readability age, difficulty score, total word count, and sentence length. This framework calculates a composite score by integrating linguistic features such as lexical difficulty, sentence length, and syntactic complexity. Specifically, the readability age means an estimate of the educational level required to understand the text and difficulty score, which means a higher score indicates more complex text.

Analysis of graded responses were performed to assess whether different chatbot and different prompt influenced scoring outcomes. Chi-square tests (χ^2^) were applied to test differences in ordinal ratings among the LLMs and between prompt conditions. For the readability and complexity metrics, one-way analysis of variance (ANOVA) tests were performed to assess differences among the three LLMs. Paired t-tests were used to compare the effects of prompt engineering within each model. Data are presented using absolute values, percentages, mean, and standard deviations (SD). All statistical procedures were performed using GraphPad Prism 9.5.1. And the level of statistical significance was set at *p* < 0.05.

## 3 Results

**Figure 1:**
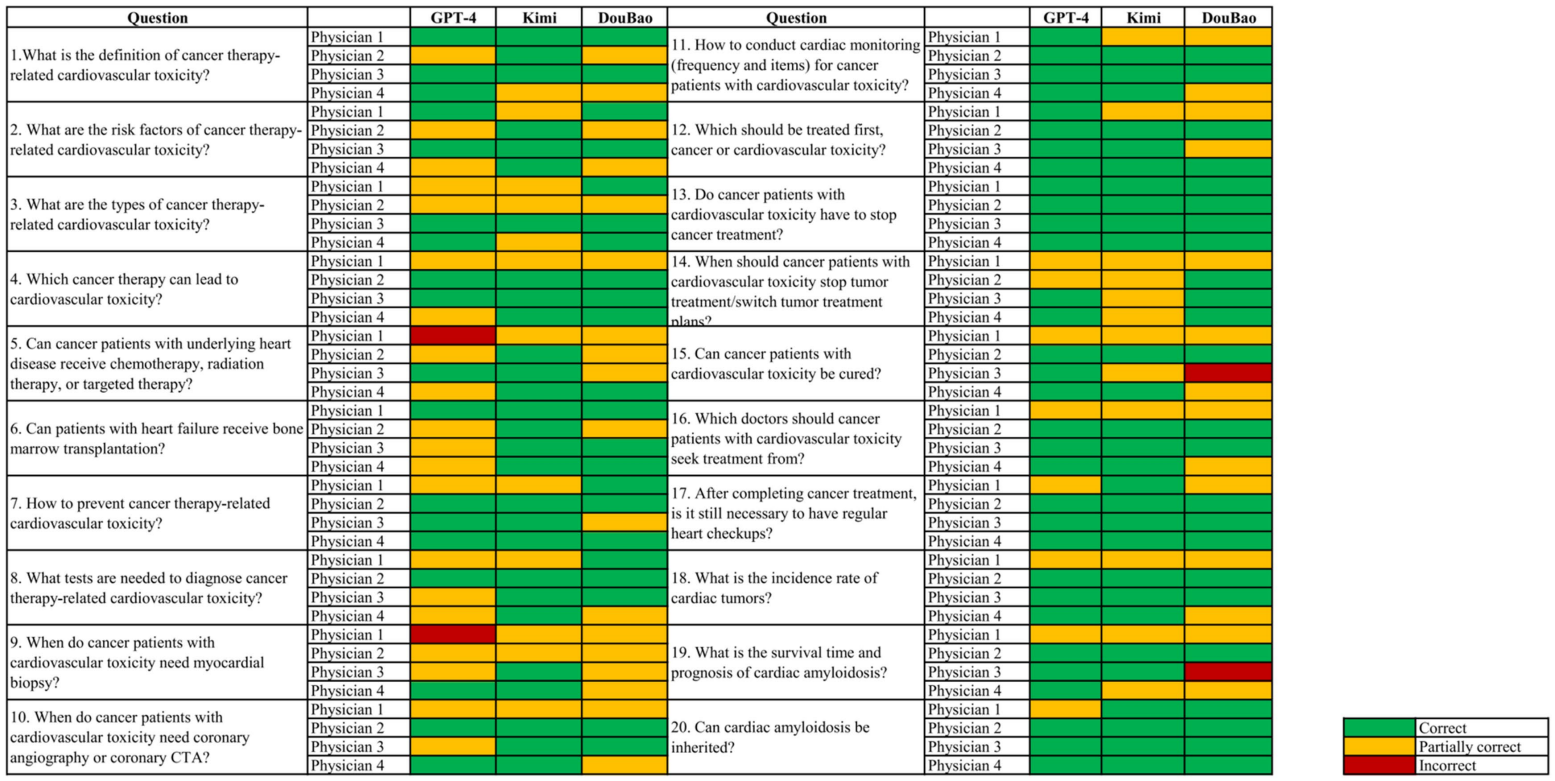
Colormap representation of the accuracy of graded responses from different LLMs.

**Figure 2:**
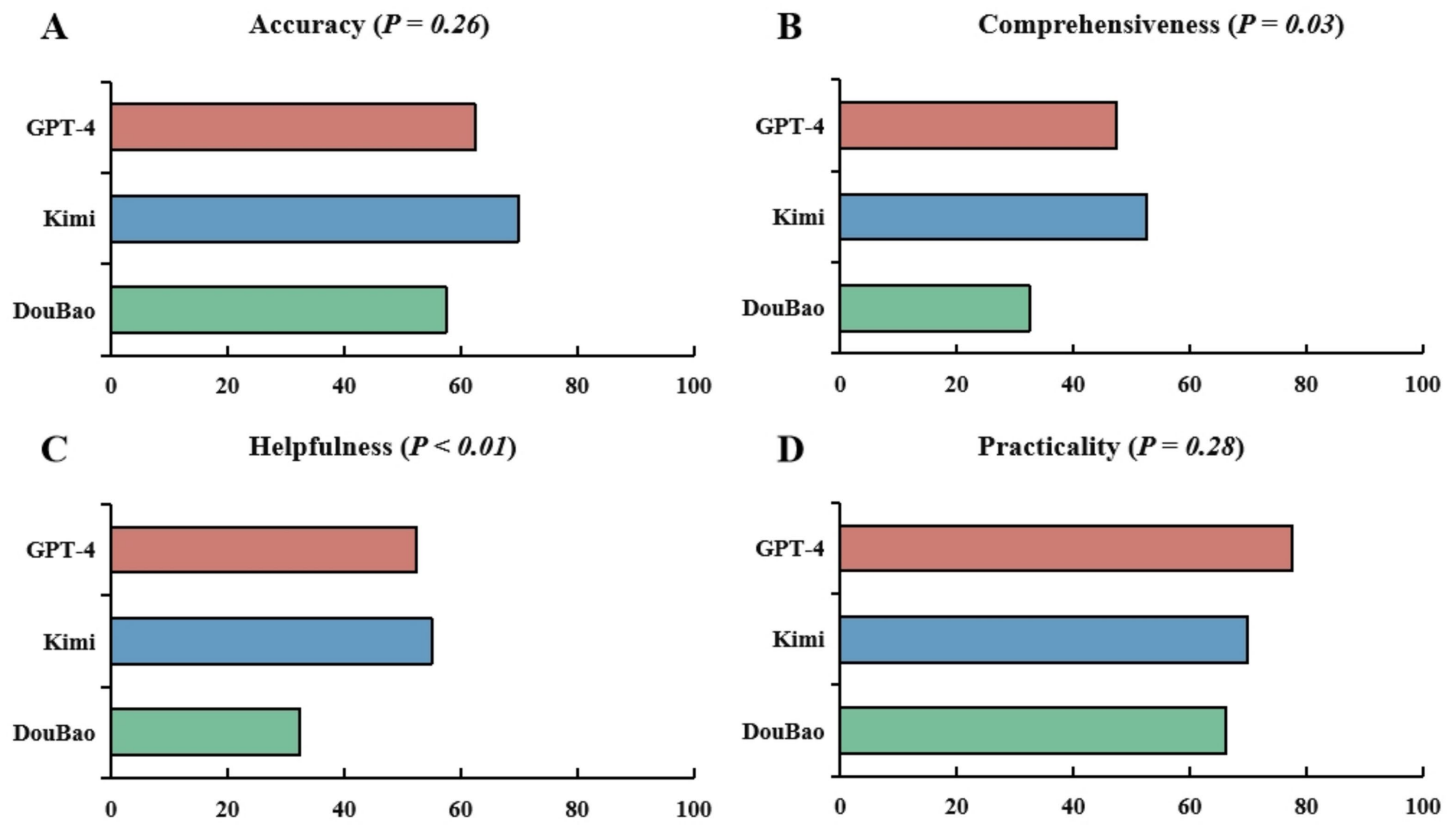
Comparative analysis of LLMs responses across four dimensions: accuracy, comprehensiveness, helpfulness, and practicality. **(A)** No significant differences were observed in response accuracy (rated as “correct”) among three LLMs (GPT-4 62.5% vs. Kimi 70% vs. DouBao 57.5%, *p* = 0.26). **(B)** There were significant differences in response comprehensiveness (rated as “comprehensive”) among three LLMs (GPT-4 47.5% vs. Kimi 52.5% vs. DouBao 32.5%, *p* = 0.03). **(C)** There were significant differences in response helpfulness (rated as “helpful”) among three LLMs (GPT-4 52.5% vs. Kimi 55% vs. DouBao 32.5%, *p* < 0.01). **(D)** No significant differences were observed in response practicality (answered “Yes” in question “*Would you use this answer for patient education?*”) among three LLMs (GPT-4 77.5% vs. Kimi 70% vs. DouBao 66.3%, *p* = 0.28).

A summative representation of three LLMs response grading in accuracy is displayed in Figure 1. The distribution of accuracy ratings for the combined question set across all three models is as follows: 63.3% (*n* = 152, 95% CI: 57.2–69.4%) of responses were rated as “correct,” 35.0% (*n* = 84, 95% CI: 29.0–41.0%) as “partially correct,” and 1.7% (*n* = 4, 95% CI: 0–3.3%) as “incorrect” (Figure 1). For GPT-4, 62.5% of responses were rated as “correct,” 35.0% as “partially correct,” and 2.5% as “incorrect.” Kimi had 70.0% of responses rated as “correct,” 30.0% as “partially correct,” and no responses were rated as “incorrect.” DouBao had 57.5% of responses rated as “correct,” 40.0% as “partially correct,” and 2.5% as “incorrect.” No statistically significant differences among LLMs were detected in the overall rating (*p* = 0.26) (Figure 2). The performance of the large language models (LLMs) across other dimensions (comprehensiveness, helpfulness, and practicality) is detailed in Table 2. Statistically significant differences were observed among the three models in comprehensiveness (*p* = 0.03) and helpfulness (*p* < 0.01) (Figure 2), while no significant difference was found in practicality (*p* = 0.28) (Figure 2).

**Table 2:** Comprehensiveness, helpfulness, and practicality ratings of three LLMs.

	Total (*N* = 240)	GPT-4 (*N* = 80)	Kimi (*N* = 80)	DouBao (*N* = 80)
Comprehensiveness				
Comprehensive	106 (44.2, 37.9–50.4)	38 (47.5, 36.6–58.4)	42 (52.5, 41.6–63.4)	26 (32.5, 22.2–42.8)
With omissions	131 (54.6, 48.3–60.9)	42 (52.5, 41.6–63.4)	37 (46.2, 35.3–57.2)	52 (65.0, 54.5–75.5)
No useful information at all	3 (1.2, 0–2.7)	0	1 (1.3, 0–3.7)	2 (2.5, 0–5.9)
Helpfulness				
Helpful	112 (46.7, 40.4–53.0)	42 (52.5, 41.6–63.4)	44 (55.0, 44.1–65.9)	26 (32.5, 22.2–42.8)
Partially helpful	111 (46.3, 39.9–52.6)	32 (40.0, 29.3–50.7)	31 (38.7, 28.1–49.4)	48 (60.0, 49.3–70.7)
Unhelpful	17 (7.1, 3.8–10.3)	6 (7.5, 1.7–13.3)	5 (6.3, 0.9–11.6)	6 (7.5, 1.7–13.3)
Practicality (*Would you use this answer for patient education?*)				
Yes	171 (71.3, 65.5–77.0)	62 (77.5, 68.3–86.7)	56 (70.0, 60.0–80.0)	53 (66.3, 55.9–76.6)
No	69 (28.7, 23.0–34.5)	18 (22.5, 13.3–31.7)	24 (30.0, 20.0–40.0)	27 (33.7, 23.4–44.1)

Data are presented as numbers (n) and percentage (%, 95 CI).

**Figure 3:**
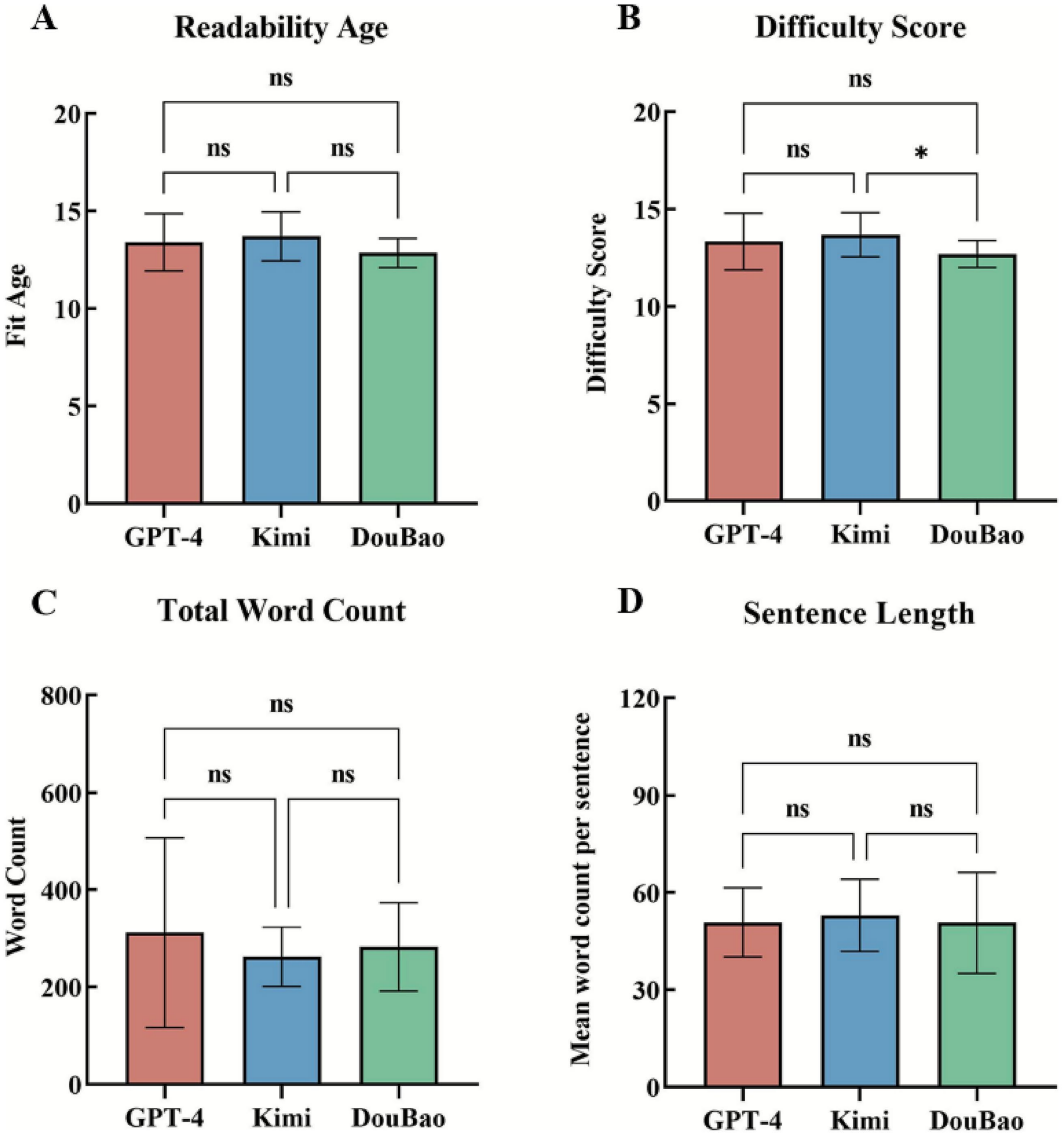
Comparison of LLMs responses in readability and complexity. **(A)** There were no significant differences in response readability age among there LLMs (GPT-4: 13.4 ± 1.5 years vs. Kimi: 13.7 ± 1.3 years vs. DouBao: 12.9 ± 0.8 years, *p* > 0.05 for all). **(B)** In response difficulty score, there were significant differences between Kimi and DouBao (13.7 ± 1.1 vs. 12.7 ± 0.7, *p* = 0.02). **(C)** There were no significant differences in the total word count of responses among there LLMs (GPT-4: 311.8 ± 195.0 vs. Kimi: 262.1 ± 70.0 vs. DouBao: 283.0 ± 90.6, *p* > 0.05 for all). **(D)** There were no significant differences in the sentence length of responses among there LLMs (GPT-4: 50.8 ± 10.6 words vs. Kimi: 53.0 ± 11.1 words vs. DouB) ao: 50.7 ± 15.6 words, *p* > 0.05 for all. means *p* < 0.05.

For readability and complexity, the three models achieved an average readability age of 13.3 ± 1.2 years, a difficulty score of 13.2 ± 1.2, a total word count of 285.6 ± 128.5, and a sentence length of 51.5 ± 12.5 words. For GPT-4, the readability age was 13.4 ± 1.5 years, the difficulty score was 13.3 ± 1.5, the total word count was 311.8 ± 195.0 and the sentence length was 50.8 ± 10.6 words. Kimi had readability age 13.7 ± 1.3 years, difficulty score 13.7 ± 1.1, total word count 262.1 ± 70.0, sentence length 53.0 ± 11.1 words. DouBao had readability age 12.9 ± 0.8 years, difficulty score 12.7 ± 0.7, total word count 283.0 ± 90.6, sentence length 50.7 ± 15.6 words. Among all metrics, only the difficulty scores between Kimi and DouBao showed a statistically significant difference (*p* = 0.02). No significant differences were observed in other readability and complexity metrics across the three models (Figure 3).

**Figure 4:**
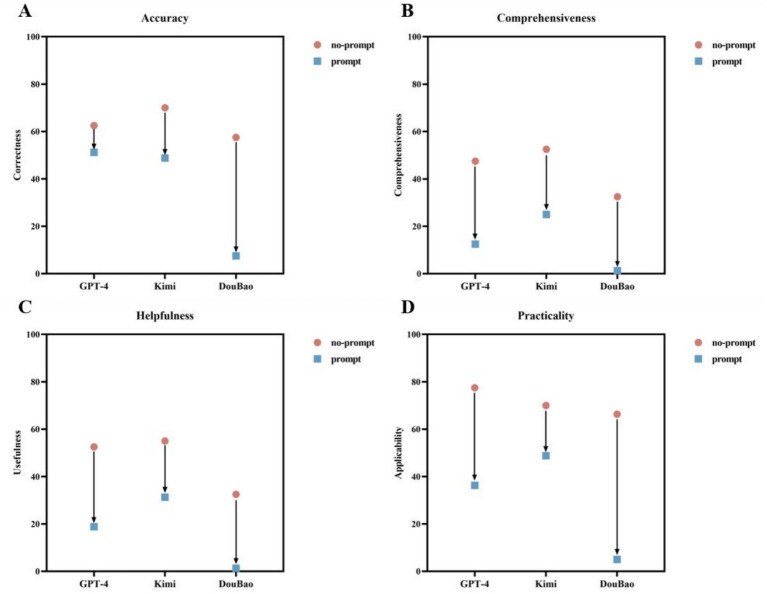
Examining the effects of prompt engineering on response accuracy, comprehensiveness, helpfulness, and practicality across three LLMs. **(A)** Prompt engineering induced significant accuracy degradation across all models, except GPT-4 (GPT-4: 62.5% vs. prompt 51.2%, *p* = 0.15; Kimi: 70.0% vs. prompt 48.8%, *p* < 0.01; DouBao: 57.5% vs. prompt 7.5%, *p* < 0.01). **(B–D)** Prompt engineering interventions resulted in statistically significant degradation across comprehensiveness, helpfulness, and practicality in the three LLMs (*Comprehensiveness*: GPT-4: 47.5% vs. prompt 12.5%, *p* < 0.01; Kimi: 52.5% vs. prompt 25.0%, *p* < 0.01; DouBao: 32.5% vs. prompt 1.3%, *p* < 0.01; *Helpfulness*: GPT-4: 52.5% vs. prompt 18.8%, *p* < 0.01; Kimi: 55.0% vs. prompt 31.3%, *p* < 0.01; DouBao: 32.5% vs. prompt 1.3%, *p* < 0.01; *Practicality*: GPT-4: 77.5% vs. prompt 36.3%, *p* < 0.01; Kimi: 70.0% vs. prompt 48.8%, *p* < 0.01; DouBao: 66.3% vs. prompt 5.0%, *p* < 0.01).

**Figure 5:**
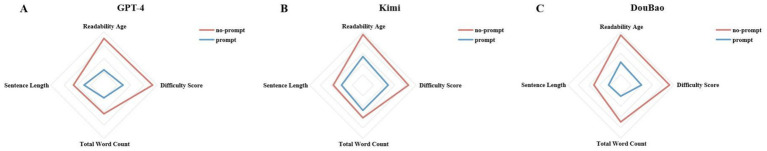
Examining the effects of prompt engineering on response readability and complexity across three LLMs. **(A)** GPT-4 had significant reductions in readability age (13.4 ± 1.5 vs. 12.2 ± 1.1 years, *p* < 0.01), difficulty score (13.3 ± 1.5 vs. 12.1 ± 1.1, *p* < 0.01), and total word count (311.8 ± 195.0 vs. 170.3 ± 60.1, *p* < 0.01), but no change in sentence length (50.8 ± 10.6 words vs. 45.2 ± 12.5 words, *p* = 0.13). **(B)** There were no significant reductions across all metrics in Kimi (*readability age*: 13.7 ± 1.3 vs. 13.2 ± 1.2 years, *p* = 0.13; *difficulty score*: 13.7 ± 1.1 vs. 13.1 ± 1.2, *p* = 0.13; *total word count*: 262.1 ± 61.0 vs. 231.2 ± 62.2, *p* = 0.12; *sentence length*: 53.0 ± 11.1 words vs. 48.1 ± 9.1 words, *p* = 0.14). **(C)** There were significant reductions across all metrics in DouBao (*readability age*: 12.9 ± 0.8 vs. 10.6{Citation} ± 1.2 years, *p* < 0.01; *difficulty score*: 12.7 ± 0.7 vs. 10.6 ± 1.1, *p* < 0.01; *total word count*: 283.0 ± 90.6 vs. 138.7 ± 28.7, *p* < 0.01; *sentence length*: 50.7 ± 15.6 words vs. 36.0 ± 9.6 words, *p* < 0.01).

Subsequently, we evaluated the impact of prompt engineering on model responses. After applying the prompt, subjective ratings for all models declined (Figure 4), but the magnitude of decline varied. In accuracy, GPT-4 exhibited the smallest decline (62.5% vs. 51.2%, *p* = 0.15), while Kimi (70.0% vs. 48.8%, *p* < 0.01) and DouBao (57.5% vs. 7.5%, *p* < 0.01) showed significant reductions. As for other dimensions, comprehensiveness, helpfulness, and practicality, statistically significant declines were observed across all models (*p* < 0.01 for all). Regarding readability and complexity, prompt engineering reduced text complexity and improved readability (Figure 5). Specifically, GPT-4 had significant reductions in readability age (13.4 ± 1.5 vs. 12.2 ± 1.1 years, *p* < 0.01), difficulty score (13.3 ± 1.5 vs. 12.1 ± 1.1, *p* < 0.01), and total word count (311.8 ± 195.0 vs. 170.3 ± 60.1, *p* < 0.01), but no change in sentence length (*p* = 0.13). As for Kimi, there were no significant reductions across all metrics (*p* > 0.12 for all). DouBao had significant reductions in readability age (12.9 ± 0.8 vs. 10.1 ± 1.2 years, *p* < 0.01), difficulty score (12.7 ± 0.7 vs. 10.6 ± 1.1, *p* < 0.01), total word count (283.0 ± 90.6 vs. 138.7 ± 28.7, *p* < 0.01), and sentence length (50.7 ± 15.6 vs. 36.0 ± 9.6 words, *p* < 0.01).

## 4 Discussion

Open-source large language models (LLMs) are advancing at an astonishing rate and have begun playing a significant role across various industries (Gibson et al., 2024; Tajiri et al., 2017; Wan et al., 2024; Li et al., 2024; Shapiro et al., 2022). However, direct application of these models in medical practice is still limited due to the complex knowledge structure, strict logical requirements, and low tolerance for error in clinical decision-making (Carl et al., 2024; Iglesias et al., 2024; Xu et al., 2020; Niraula et al., 2023). The field of cardio-oncology, an emerging interdisciplinary area, has a huge demand for medical public education content (Tajiri et al., 2017). The use of LLMs to generate medical education materials can help bridge the information gap between healthcare providers and patients, providing high-quality, easily understandable content for non-medical audiences (An et al., 2024). Moreover, AI-generated patient education and informational content carry relatively low risk, as it can be reviewed by medical professionals, ensuring higher tolerance for error.

In this study, we designed 20 questions related to cardio-oncology, covering fundamental topics such as tumor-related heart diseases, prevention, diagnosis, treatment, and even heart tumors. These questions varied in complexity, with some being open-ended, allowing us to evaluate the models’ performance in terms of accuracy, comprehensiveness, helpfulness, practicality and so on.

Accuracy is the most critical metric for assessing model performance. In this study, most of the models’ answers were judged as “correct” or “partially correct.” Statistical analysis revealed no significant difference in the accuracy rates of the three models, with Kimi generating no completely incorrect responses. In terms of other subjective evaluation indicators, DouBao exhibited weaker performance in terms of “comprehensiveness” and “helpfulness” as it occasionally produced responses that were correct but lacked practical guidance. This suggests that accuracy is not the only evaluation criterion, and it is necessary to establish an expert system evaluation framework for medical education that integrates general evaluation methods for LLMs.

Notably, when the simple prompt “*Please answer the following question in the most straightforward and easy-to-understand language*” was added to all questions, subjective evaluation scores for all models decreased, with DouBao showing the most significant decline. This indicates that a single, simple prompt strategy may reduce the quality of responses to complex medical questions. This phenomenon may be related to the text generation mechanism of LLMs, when add the prompt (“*Please answer the following question in the most straightforward and easy-to-understand language*”), the LLMs will give priority to simplifying the answer and tend to use non-professional terms. However, in this specialized medical context, the omitted content is often the key to ensuring the informational accuracy. Consequently, the simplification process may lead to the loss of necessary clinical context, thereby diminishing the professionalism of the model’s responses. Additionally, it is important to note that in public chat interfaces, users generally cannot directly adjust key model hyperparameters such as temperature, maximum token count, top-p, or repetition penalties, which are typically configured only through API calls or specialized environments (Joseph et al., 2024). Therefore, our findings reflect the performance of these models under their default, “out-of-the-box” settings. This underscores the need to develop and optimize dedicated expert models or tailor fine-tuning protocols for specific medical scenarios. Future efforts could draw on successful transfer learning strategies used to optimize domain-specific models in other fields (Eralp and Sefer, 2024).

Regarding the objective evaluation system, we applied Chinese text analysis framework, including readability and complexity, to evaluate the models based on four dimensions: readability age, difficulty score, total word count, and sentence length. No clear differences were found in the objective performance of the three LLMs. However, after adding the prompt, the performance of the three LLMs declined across all four dimensions. These findings highlight a significant discrepancy between objective and subjective evaluations, suggesting that current objective evaluation tools are relatively basic and cannot fully replace subjective evaluations. Thus, more specialized objective evaluation methods should be developed to provide a solid foundation for the automatic evaluation of LLM responses.

This study has certain limitations. Firstly, despite using a blind method and multiple expert reviewers, subjective evaluations may still have some bias, and the study did not include patient feedback on the model-generated content. Additionally, the question pool, while based on clinical guidelines and expert input, was limited in size and may not cover all aspects of cardio-oncology. Future studies would benefit from a larger, formally validated question set. As large model versions continue to evolve, further research is needed to address the evidence-based challenges of using LLMs in medical scenarios. Furthermore, our outcome measures were based on expert ratings. Future studies with a validated question-answer benchmark could employ standard AI performance metrics such as F1-score. Finally, while our study evaluated only three LLMs, the rapidly evolving landscape means that other models, such as DeepSeek, which focuses on strong reasoning capabilities, were not included. Future studies could benefit from incorporating a more diverse array of models to provide a comprehensive performance landscape.

## 5 Conclusion

This study evaluates the application value of three different types of LLMs in the field of cardio-oncology. The results indicate that most models provide accurate responses, but careful prompt design and more detailed parameter fine-tuning are necessary to better serve clinical applications. The findings offer valuable insights and data for the design and evaluation of medical professional models. We look forward to more evaluation studies in the future and hope that public models will develop more expert-level versions or further tuning to meet the needs of real-world patient education scenarios.

## Data Availability

The raw data supporting the conclusions of this article will be made available by the authors, without undue reservation.
