# Supplementary material for: Evaluating the efficacy of large language models in cardio-oncology patient education: a comparative analysis of accuracy, readability, and prompt engineering strategies
Source: Front Artif Intell. 2026 Jan 13;8:1693446. doi: 10.3389/frai.2025.1693446 (PMC12835249; doi:10.3389/frai.2025.1693446)
Supplement: Supplementary file 1 [file Data_Sheet_1.pdf]

Figure S1 Colormap representation of the comprehensiveness of graded responses from different LLMs.

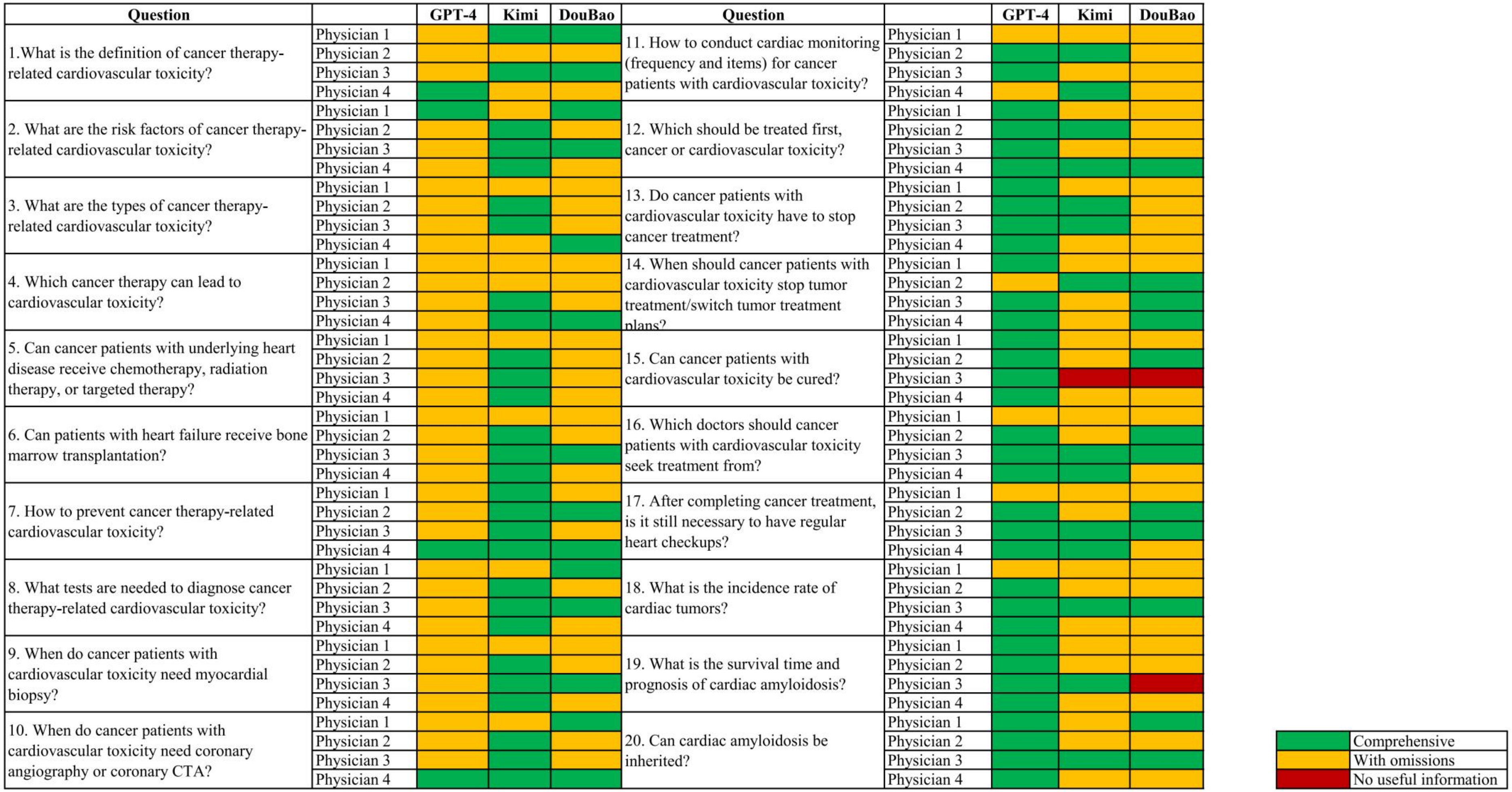

Figure S2 Colormap representation of the helpfulness of graded responses from different LLMs.

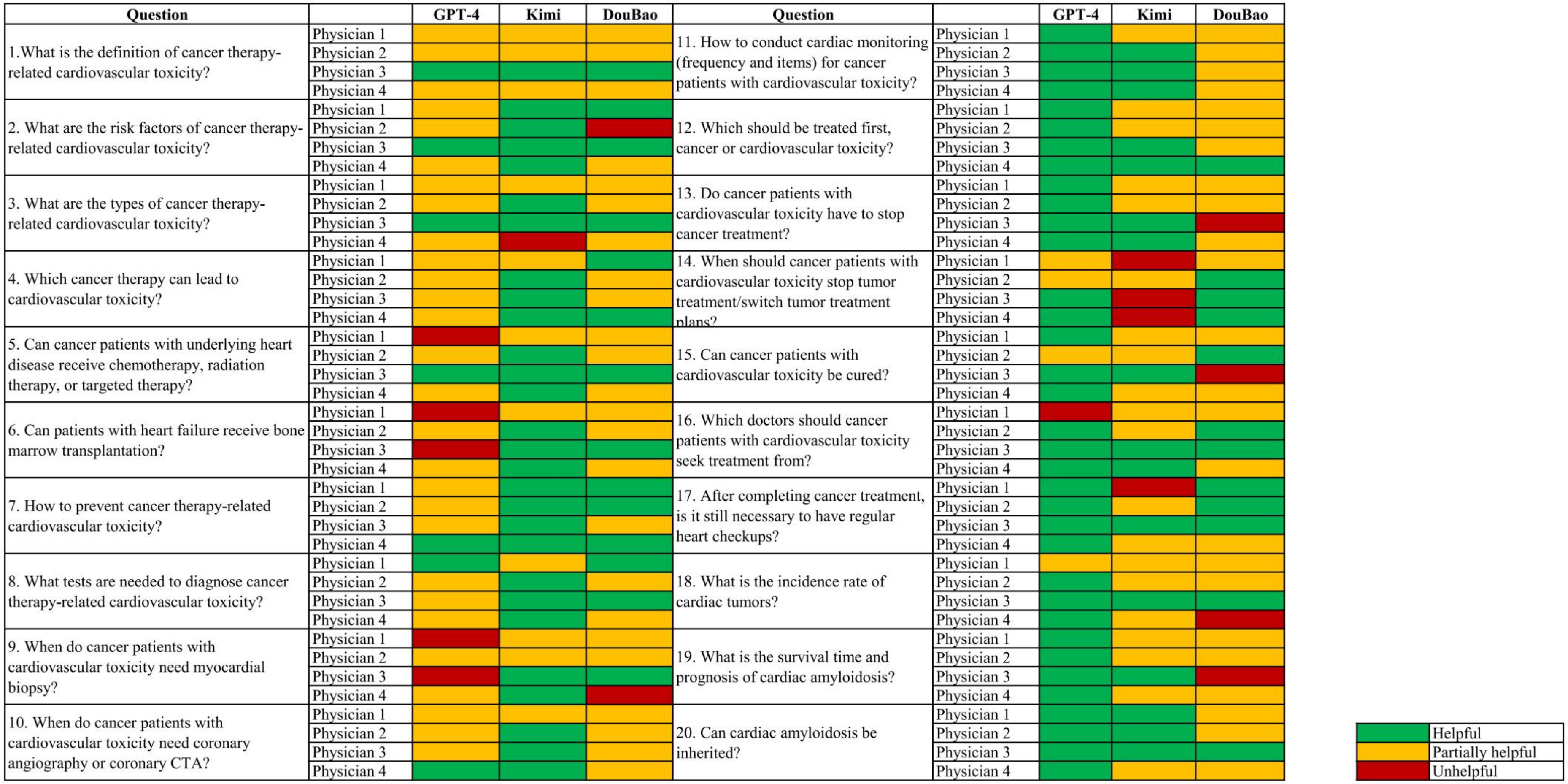

Figure S3 Colormap representation of the practicality of graded responses from different LLMs.

Question		GPT-4	Kimi	DouBao
1.What is the definition of cancer therapy-related cardiovascular toxicity?	Physician 1	No	No	Yes
	Physician 2	Yes	No	No
	Physician 3	Yes	Yes	Yes
	Physician 4	Yes	No	Yes
2. What are the risk factors of cancer therapy-related cardiovascular toxicity?	Physician 1	Yes	No	Yes
	Physician 2	Yes	Yes	No
	Physician 3	Yes	Yes	Yes
	Physician 4	Yes	Yes	No
3. What are the types of cancer therapy-related cardiovascular toxicity?	Physician 1	No	No	Yes
	Physician 2	No	Yes	Yes
	Physician 3	Yes	Yes	Yes
	Physician 4	No	No	Yes
4. Which cancer therapy can lead to cardiovascular toxicity?	Physician 1	No	Yes	Yes
	Physician 2	Yes	Yes	Yes
	Physician 3	No	Yes	No
	Physician 4	Yes	Yes	Yes
5. Can cancer patients with underlying heart disease receive chemotherapy, radiation therapy, or targeted therapy?	Physician 1	No	Yes	No
	Physician 2	No	Yes	No
	Physician 3	No	Yes	Yes
	Physician 4	Yes	Yes	No
6. Can patients with heart failure receive bone marrow transplantation?	Physician 1	No	Yes	Yes
	Physician 2	No	Yes	Yes
	Physician 3	No	Yes	Yes
	Physician 4	No	Yes	Yes
7. How to prevent cancer therapy-related cardiovascular toxicity?	Physician 1	Yes	Yes	Yes
	Physician 2	Yes	No	Yes
	Physician 3	Yes	Yes	Yes
	Physician 4	Yes	Yes	Yes
8. What tests are needed to diagnose cancer therapy-related cardiovascular toxicity?	Physician 1	Yes	No	Yes
	Physician 2	Yes	Yes	No
	Physician 3	Yes	Yes	Yes
	Physician 4	No	Yes	No
9. When do cancer patients with cardiovascular toxicity need myocardial biopsy?	Physician 1	No	Yes	Yes
	Physician 2	Yes	Yes	No
	Physician 3	Yes	Yes	Yes
	Physician 4	Yes	Yes	No
10. When do cancer patients with cardiovascular toxicity need coronary angiography or coronary CTA?	Physician 1	Yes	Yes	Yes
	Physician 2	Yes	Yes	Yes
	Physician 3	No	Yes	Yes
	Physician 4	Yes	Yes	No

Question		GPT-4	Kimi	DouBao
11. How to conduct cardiac monitoring (frequency and items) for cancer patients with cardiovascular toxicity?	Physician 1	Yes	No	Yes
	Physician 2	Yes	Yes	No
	Physician 3	Yes	Yes	No
	Physician 4	Yes	Yes	Yes
12. Which should be treated first, cancer or cardiovascular toxicity?	Physician 1	Yes	No	No
	Physician 2	Yes	No	Yes
	Physician 3	Yes	Yes	No
	Physician 4	Yes	Yes	Yes
13. Do cancer patients with cardiovascular toxicity have to stop cancer treatment?	Physician 1	Yes	No	No
	Physician 2	Yes	Yes	Yes
	Physician 3	Yes	Yes	Yes
	Physician 4	Yes	Yes	Yes
14. When should cancer patients with cardiovascular toxicity stop tumor treatment/switch tumor treatment plans?	Physician 1	Yes	No	Yes
	Physician 2	No	Yes	Yes
	Physician 3	Yes	No	No
	Physician 4	Yes	No	Yes
15. Can cancer patients with cardiovascular toxicity be cured?	Physician 1	Yes	Yes	Yes
	Physician 2	Yes	No	Yes
	Physician 3	Yes	Yes	No
	Physician 4	Yes	No	No
16. Which doctors should cancer patients with cardiovascular toxicity seek treatment from?	Physician 1	Yes	Yes	No
	Physician 2	Yes	No	Yes
	Physician 3	Yes	Yes	Yes
	Physician 4	Yes	Yes	No
17. After completing cancer treatment, is it still necessary to have regular heart checkups?	Physician 1	Yes	Yes	Yes
	Physician 2	Yes	Yes	Yes
	Physician 3	Yes	Yes	Yes
	Physician 4	Yes	No	Yes
18. What is the incidence rate of cardiac tumors?	Physician 1	No	Yes	No
	Physician 2	Yes	No	Yes
	Physician 3	Yes	Yes	Yes
	Physician 4	Yes	No	No
19. What is the survival time and prognosis of cardiac amyloidosis?	Physician 1	Yes	Yes	Yes
	Physician 2	Yes	Yes	Yes
	Physician 3	Yes	No	Yes
	Physician 4	Yes	No	No
20. Can cardiac amyloidosis be inherited?	Physician 1	Yes	Yes	Yes
	Physician 2	Yes	Yes	No
	Physician 3	Yes	Yes	Yes
	Physician 4	Yes	No	No

(green)	Yes
(orange)	No
